# Identification of Growth-Related Single-Nucleotide Polymorphisms in the cAMP-Dependent Protein Kinase Type I Regulatory Subunit-like Gene of the Oriental River Prawn, *Macrobrachium nipponense*

**DOI:** 10.3390/ijms27167500

**Published:** 2026-08-21

**Authors:** Yuefan Zhang, Hao Dong, Xiaofan Fang, Hui Qiao, Wenyi Zhang, Yiwei Xiong, Sufei Jiang, Shubo Jin

**Affiliations:** 1 Key Laboratory of Mariculture & Stock Enhancement in North China’s Sea, Ministry of Agriculture and Rural Affairs, Dalian Ocean University, Dalian 116023, China; 2 Key Laboratory of Freshwater Fisheries and Germplasm Resources Utilization, Ministry of Agriculture and Rural Affairs, Freshwater Fisheries Research Center, Chinese Academy of Fishery Sciences, Wuxi 214081, China; 3 Wuxi Fisheries College, Nanjing Agricultural University, Wuxi 214081, China

**Keywords:** *Macrobrachium nipponense*, *Mn-PKA-R1*, single nucleotide polymorphism, growth-related traits, association analysis, marker-assisted selection

## Abstract

*Macrobrachium nipponense* is an economically important freshwater crustacean in China, and growth-related traits are major determinants of its commercial value. Identifying single nucleotide polymorphisms (SNPs) associated with growth-related traits may provide useful molecular markers for the genetic improvement of growth performance. Previous studies have suggested that the cAMP-dependent protein kinase type I regulatory subunit-like gene (*Mn-PKA-R1*) is involved in the regulation of growth and molting in *M. nipponense*. In the present study, a cultured full-sib family comprising 120 females and 120 males was used to screen SNPs within the coding region of *Mn-PKA-R1*, and evaluate their associations with body weight, body length, full length, and abdominal width. Seven SNPs were identified in exon 12, including five nonsynonymous missense variants and two synonymous variants, whereas no SNPs were detected in the other exons examined. The mean effective number of alleles (*N_e_*), observed heterozygosity (*H_o_*), expected heterozygosity (*H_e_*), Nei’s gene diversity (Nei), and polymorphic information content (PIC) were 1.3561, 0.3048, 0.2350, 0.2340, and 0.1962, respectively, in females, compared with 1.3118, 0.2714, 0.1868, 0.1861, and 0.1512, respectively, in males. Four SNPs were significantly associated with growth-related traits in females: C+82748G and A+82750G were associated with body weight and abdominal width, T+82810G was associated with abdominal width, and A+83324G was associated with body weight, full length, and abdominal width (*p* < 0.05). No significant genotype–trait associations were detected among the SNP loci that could be statistically evaluated in males. Pairwise *r*^2^ values ranged from 0.001 to 0.693 in females and from 0.000 to 0.351 in males, with generally higher values observed in females within the investigated full-sib family. Females also showed slightly higher mean genetic polymorphism parameters than males, while significant SNP–trait associations were detected only in females within this family. These findings provide candidate SNPs for further validation of their potential utility in marker-assisted selection in *M. nipponense*.

## 1. Introduction

*Macrobrachium nipponense*, commonly known as the oriental river prawn, is an economically important freshwater crustacean in China [1]. In 2025, the aquaculture production of *M. nipponense* reached 227,687 metric tons, ranking fourth among the major shrimp species farmed in freshwater in China [2]. Its main aquaculture regions are concentrated in eastern and southeastern China, particularly in Jiangsu, Anhui, and Zhejiang provinces [2]. This species is characterized by fast growth [3], broad environmental adaptability [4,5], and strong market demand [1], making it one of the most commercially valuable species in Chinese freshwater aquaculture. Notably, *M. nipponense* exhibits pronounced sexual dimorphism in growth, with males generally growing faster and attaining larger body sizes at harvest than females [6].

Growth-related traits, including body weight, body length, full length, and abdominal width, are primary determinants of commercial value and aquaculture productivity, and therefore represent principal target traits in selective breeding programs [7]. Improving growth performance can shorten the production cycle [8], improve feed utilization [9], and contribute to aquaculture productivity [10].

In recent three decades, three new varieties of *M. nipponense*, named “Taihu No. 1”, “Taihu No. 2”, and “Taihu No. 3”, have been developed through genetic improvement of traditional techniques by Freshwater Fisheries Research Center, Chinese Academy of Fishery Sciences, with body weight as the target trait. These three varieties have been approved as new varieties by the Ministry of Agriculture and Rural Affairs. Traditional selective breeding primarily relies on phenotypic records and may be constrained by long generation intervals, environmental effects on phenotypic expression, and the costs associated with large-scale phenotyping [11,12]. These constraints collectively underscore the urgent need for more efficient, genetically informed breeding strategies. In contrast, marker-assisted breeding offers a more precise and efficient approach [13,14]. Identifying SNPs associated with growth-related traits can provide candidate molecular markers for subsequent validation and breeding applications [15].

Among the diverse array of molecular markers currently available, SNPs have emerged as one of the most extensively utilized and analytically powerful marker types in contemporary genomic research [16]. Their application in aquatic animal breeding has demonstrated considerable promise in facilitating the genetic improvement of economically important traits, including growth performance and disease resistance, providing a robust theoretical and empirical foundation for the development of high-quality, selectively bred aquatic species [17]. In recent years, SNP screening has been increasingly applied to investigate the genetic basis of economically important traits in *M. nipponense*, yielding a growing body of molecular resources for breeding improvement. Early efforts focused on candidate gene approaches, in which SNPs within functionally characterized genes were identified and assessed for associations with target traits. For instance, SNP loci within the glutathione S-transferase-2 (*GST-2*) gene were identified, and found to be significantly associated with both hypoxia tolerance and growth traits, including full length, body weight, and body length, across multiple *M. nipponense* populations, suggesting their potential utility as molecular markers for simultaneously improving stress tolerance and growth performance [18]. SNP screening of the *Mn-CTSL1* identified multiple loci significantly associated with growth traits in both male and female *M. nipponense*, with certain candidate loci showing positive associations with superior growth performance, and others proposed as potential molecular markers for marker-assisted selection aimed at improving growth and gonadal development traits in *M. nipponense* [19]. SNP loci significantly associated with growth-related traits were identified in the *ACTL* gene of *M. nipponense* [20].

Previous RNA interference studies have shown that *Mn-PKA-R1* is involved in the regulation of growth and molting in *M. nipponense* [21]. Based on this evidence, the present study screened SNPs within the coding region of *Mn-PKA-R1* in a cultured full-sib family using polymerase chain reaction (PCR) amplification and sequencing and subsequently evaluated their associations with growth-related traits. The SNPs showing associations with growth-related traits in this family represent potential candidate markers for further evaluation in marker-assisted selection in *M. nipponense*.

## 2. Results

### 2.1. Gene Structure

**Figure 1:**
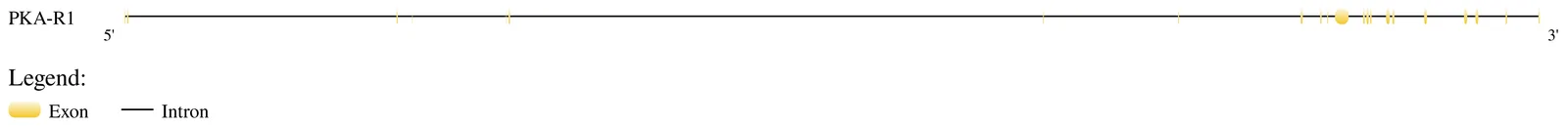
Genomic structure of the *Mn-PKA-R1* gene.

The open reading frame of *Mn-PKA-R1* was 3345 bp in length, encoding a predicted protein of 1114 amino acids, and was mapped to chromosome 4 at nucleotide positions 128,346,744–128,443,339 (Figure 1). The genomic sequence of *Mn-PKA-R1* was 96,596 bp in length and comprised 22 exons and 21 introns (Table 1).

### 2.2. SNP Polymorphism Analysis of the Mn-PKA-R1 Gene

Analysis of the *Mn-PKA-R1* sequences identified seven high-quality SNP loci among the 240 *M. nipponense* individuals. These loci were named according to the nucleotide position of each variant relative to the start codon (ATG) in the genomic sequence. Secondary peaks with heights less than 10% of the major peak were considered sequencing noise and excluded. A total of seven high-confidence SNPs were detected (Table 2), all of which were located in exon 12 of *Mn-PKA-R1*. No SNPs were detected in the other exons examined. Functional annotation showed that five SNPs were nonsynonymous missense variants, whereas two were synonymous variants. Specifically, C+82748G, A+82750G, T+82768G, T+82810G, and A+83142G resulted in the amino acid substitutions p.Asp312Glu, p.Gln313Arg, p.Val319Gly, p.Val333Gly, and p.Asn444Asp, respectively. In contrast, A+82766G and A+83324G did not alter the encoded amino acids, and were classified as synonymous variants. The genetic polymorphism parameters of the seven SNP loci were analyzed separately for female individuals, male individuals, and the combined sample using PopGene32 (Table 3, Table 4 and Table 5). Among female individuals, the *N_e_* ranged from 1.0689 to 1.7909, with a mean value of 1.3561, whereas among male individuals, it ranged from 1.0084 to 1.9406, with a mean value of 1.3118. In the combined sample, *Ne* ranged from 1.0382 to 1.8749, with a mean value of 1.3339. The average *H_o_*, *H_e_*, Nei, and PIC were 0.3048, 0.2350, 0.2340, and 0.1962, respectively, in females, compared with 0.2714, 0.1868, 0.1861, and 0.1512, respectively, in males. The corresponding values in the combined sample were 0.2881, 0.2131, 0.2127, and 0.1771, respectively. The PIC values ranged from 0.0624 to 0.3441 in females, from 0.0083 to 0.3672 in males, and from 0.0361 to 0.3578 in the combined sample.

### 2.3. Phenotypic Correlations and SNP–Trait Associations

**Figure 2:**
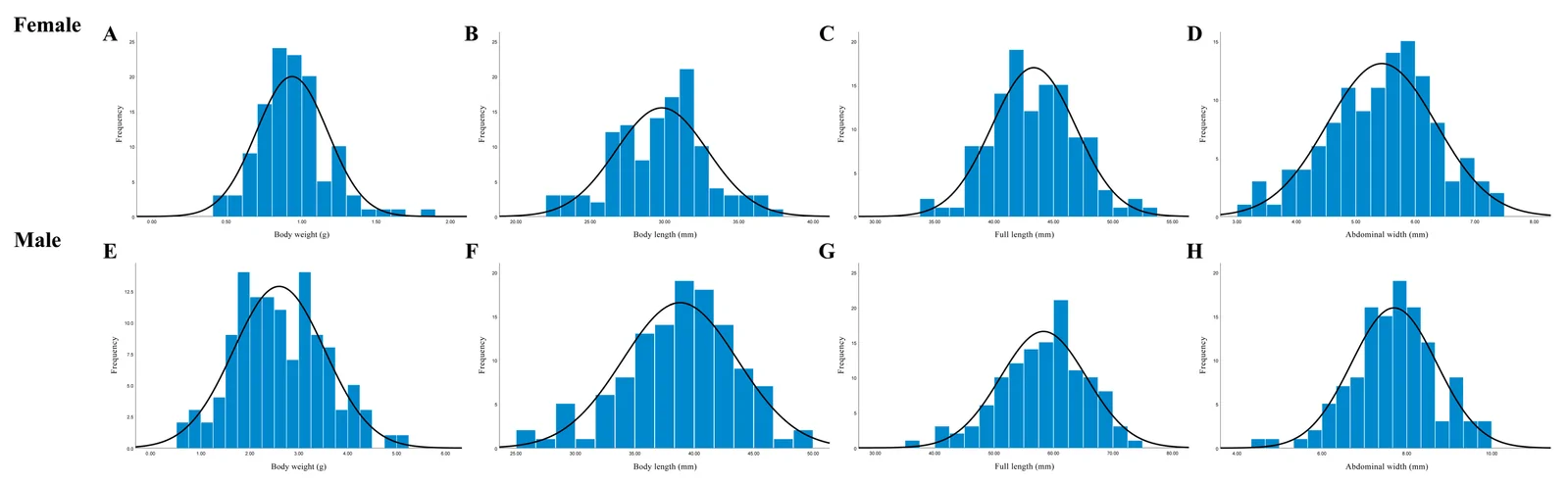
Frequency distributions of growth-related traits in female and male *M. nipponense*. (**A**–**D**) Body weight, body length, full length, and abdominal width in female individuals, respectively; (**E**–**H**) body weight, body length, full length, and abdominal width in male individuals, respectively. The black solid curves represent fitted normal distribution curves.

The Kolmogorov–Smirnov test showed that the *p*-values for body weight, body length, full length, and abdominal width were 0.092, ≥0.200, ≥0.200, and ≥0.200, respectively, in female individuals, and 0.163, ≥0.200, ≥0.200, and ≥0.200, respectively, in male individuals. Since all *p*-values were greater than 0.05, the four growth-related traits in both female and male individuals were considered to be approximately normally distributed (Figure 2).

**Figure 3:**
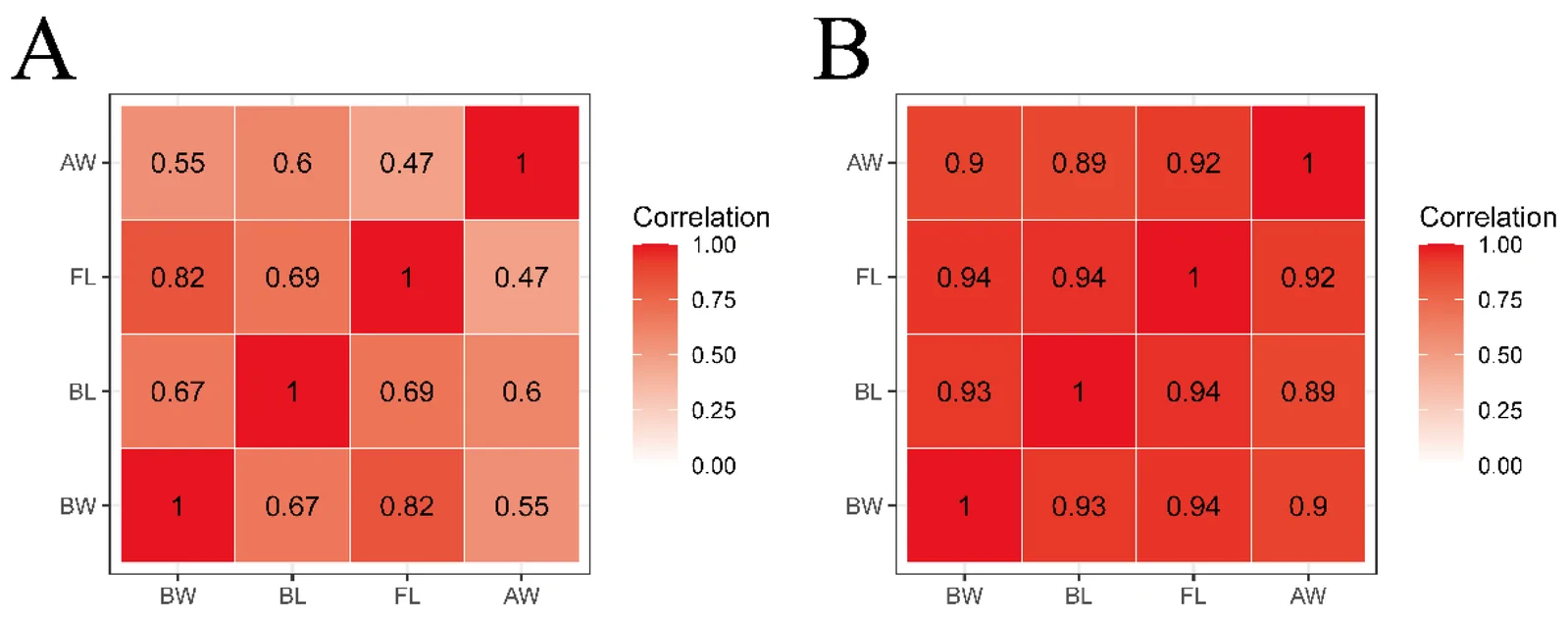
Pearson correlation heatmaps of growth-related traits in female and male *M. nipponense*. (**A**) Female individuals; (**B**) male individuals. BW, body weight; BL, body length; FL, full length; AW, abdominal width. The values in the cells represent Pearson correlation coefficients.

The correlation coefficients among growth-related traits in females ranged from 0.472 to 0.825. The strongest correlation was observed between body weight and full length (*r* = 0.825), followed by body length and full length (*r* = 0.686) and body weight and body length (*r* = 0.673). The weakest correlation was observed between full length and abdominal width (*r* = 0.472). In males, the correlation coefficients among growth-related traits ranged from 0.890 to 0.940. The strongest correlation was observed between body length and full length (*r* = 0.940), followed by body weight and full length (*r* = 0.935) and body weight and body length (*r* = 0.929). The correlation between body length and abdominal width was relatively lower (*r* = 0.890), but remained strongly positive (Figure 3).

Associations between all seven SNP loci and growth-related traits were evaluated separately in females and males (Table 6). Significant associations were detected at four loci in females, whereas no significant associations were detected among the SNP loci that could be statistically evaluated in males. At the C+82748G and A+82750G loci, female individuals carrying the CC and AA genotypes, respectively, exhibited significantly greater body weight and abdominal width than those carrying the corresponding heterozygous genotypes, CG and AG (*p* < 0.05). No significant differences were detected in body length or full length between genotypes at either locus. At the T+82810G locus, female individuals with the TT genotype exhibited significantly greater abdominal width than those with the TG genotype (*p* < 0.05), whereas body weight, body length, and full length did not differ significantly between genotypes. At the A+83324G locus, female individuals with the AA genotype exhibited significantly greater body weight, full length, and abdominal width than those with the AG genotype (*p* < 0.05), whereas no significant difference was detected in body length. In male individuals, no significant associations with growth-related traits were detected for the SNP loci that could be statistically evaluated.

### 2.4. LD Analysis of Mn-PKA-R1 SNPs

**Figure 4:**
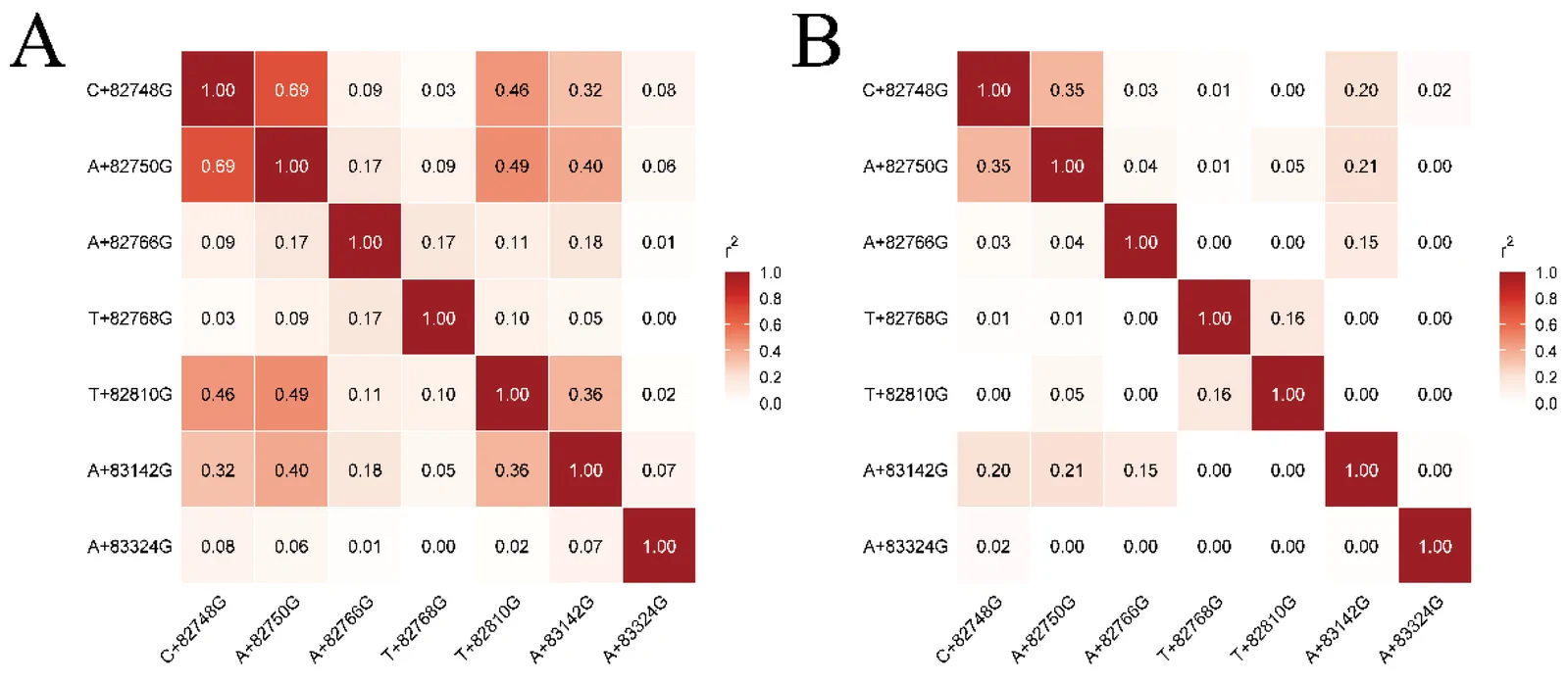
Pairwise linkage disequilibrium (*r*^2^) among the seven SNP loci of the *Mn-PKA-R1* gene in female (**A**) and male (**B**) *M. nipponense*. The color intensity represents the magnitude of pairwise linkage disequilibrium, with darker red indicating higher *r*^2^ values.

Pairwise linkage disequilibrium (LD) among the seven SNP loci of the *Mn-PKA-R1* gene was evaluated separately in female and male *M. nipponense* using the squared correlation coefficient (*r*^2^) (Figure 4).

Among female individuals, pairwise LD values ranged from 0.001 to 0.693. The highest *r*^2^ value was observed between C+82748G and A+82750G (*r*^2^ = 0.693), followed by A+82750G–T+82810G (*r*^2^ = 0.486), C+82748G–T+82810G (*r*^2^ = 0.458), A+82750G–A+83142G (*r*^2^ = 0.403), and T+82810G–A+83142G (*r*^2^ = 0.359). Most of the remaining SNP pairs had *r*^2^ values below 0.20.

Among male individuals, pairwise LD values ranged from 0.000 to 0.351. The highest *r*^2^ value was also observed between C+82748G and A+82750G (*r*^2^ = 0.351), followed by A+82750G–A+83142G (*r*^2^ = 0.206), C+82748G–A+83142G (*r*^2^ = 0.203), and T+82768G–T+82810G (*r*^2^ = 0.163). Most of the remaining SNP pairs had *r*^2^ values below 0.10.

## 3. Discussion

Protein kinase A (PKA) is a highly conserved serine/threonine kinase and a major intracellular effector of the cyclic adenosine monophosphate (cAMP) signaling pathway [22,23]. In *M. nipponense*, *Mn-PKA-R1* has been implicated in the regulation of growth and molting, suggesting its potential involvement in physiological development [21]. The present study aimed to identify SNP loci within the *Mn-PKA-R1* gene in a cultured full-sib family and evaluate their associations with growth-related traits in female and male *M. nipponense*.

*Mn-PKA-R1* spanned approximately 96.6 kb, and consisted of 22 exons and 21 introns. Seven SNP loci were identified in exon 12, and subsequently evaluated through genetic polymorphism, association, and linkage disequilibrium analyses. Five SNPs were classified as nonsynonymous missense variants, whereas two were synonymous variants. Although nonsynonymous variants alter the encoded amino acid sequence, not all amino acid substitutions affect protein structure or function; therefore, their biological effects require further computational prediction and experimental validation [24,25].

All seven SNP loci were polymorphic in the female, male, and combined samples. The mean *N_e_*, *H_o_*, *H_e_*, Nei, and PIC values were 1.3561, 0.3048, 0.2350, 0.2340, and 0.1962, respectively, in females; 1.3118, 0.2714, 0.1868, 0.1861, and 0.1512, respectively, in males; and 1.3339, 0.2881, 0.2131, 0.2127, and 0.1771, respectively, in the combined sample. These parameters were generally slightly higher in females than in males, whereas the combined sample exhibited intermediate values. Under the present single-family design, these differences should be interpreted as variation in within-family polymorphism parameters rather than as differences in population-level genetic diversity. The *H_o_* was generally higher than the *H_e_* in all three sample groups, which may reflect the limited number of parents and the specific parental allele combinations in this single full-sib family rather than population-level processes [26,27]. Several SNP loci lacked one of the possible homozygous genotypes in the analyzed offspring. Because all individuals were derived from a single full-sib family, this pattern may be related to the parental allele combinations and segregation within this family. However, the parental individuals were not genotyped for these SNP loci in the present study; therefore, the parental genotype combinations and the exact segregation patterns cannot be determined. According to the PIC classification criteria [28], loci with PIC < 0.25 are considered to exhibit low polymorphism, those with 0.25 ≤ PIC < 0.50 exhibit moderate polymorphism, and those with PIC ≥ 0.50 exhibit high polymorphism. PIC values ranged from 0.0624 to 0.3441 in females, from 0.0083 to 0.3672 in males, and from 0.0361 to 0.3578 in the combined sample. In females, C+82748G, A+82750G, and T+82810G exhibited moderate polymorphism, with PIC values of 0.3441, 0.3236, and 0.2546, respectively. In males, C+82748G and A+82750G showed moderate polymorphism, with corresponding PIC values of 0.3672 and 0.3457, whereas their PIC values in the combined sample were 0.3578 and 0.3356, respectively. The remaining loci exhibited low polymorphism, and no highly polymorphic loci were detected. Nevertheless, low or moderate PIC values do not preclude SNPs from being associated with economically important traits [29,30]. Therefore, PIC should not be used as the sole criterion for evaluating the breeding potential of a SNP; its association with economically important traits and its validation in independent families and populations should also be considered.

Four *Mn-PKA-R1* SNP loci were significantly associated with one or more growth-related traits, and all significant associations were detected in females. C+82748G and A+82750G were each associated with body weight and abdominal width, T+82810G was associated with abdominal width, and A+83324G was associated with body weight, full length, and abdominal width. Pearson correlation analysis showed that all four growth-related traits were positively correlated, with correlation coefficients ranging from 0.472 to 0.825 in females and from 0.890 to 0.940 in males, indicating coordinated variation among these growth characteristics. Accordingly, C+82748G and A+82750G were each associated with two growth-related traits, whereas A+83324G was associated with three traits. These multi-trait associations may reflect shared genetic components or biological pathways involved in overall growth performance [31]. Although growth is a complex quantitative trait influenced by multiple genes and environmental factors, the consistent associations observed across several correlated traits support the further evaluation of these loci as candidate markers for growth-related selection [32]. Among the four growth-associated loci, C+82748G, A+82750G, and T+82810G were nonsynonymous missense variants, whereas A+83324G was synonymous. The association observed at the synonymous A+83324G locus may result from linkage disequilibrium with an uncharacterized functional variant; however, a direct regulatory effect of this synonymous substitution cannot be excluded [33,34]. The association observed at A+83324G should be interpreted with particular caution because only nine female individuals carried the AG genotype, whereas 111 carried the AA genotype. The relatively small number of AG individuals may reduce the precision and generalizability of the estimated genotype effect. Therefore, this locus should be considered a candidate growth-associated SNP requiring further validation. No significant genotype–trait associations were detected among the SNP loci that could be statistically evaluated in males. The female-only SNP–trait associations should be interpreted cautiously. Although males exhibited higher absolute growth-related trait values, this does not necessarily imply stronger genotype–trait associations within males. Likewise, the higher genetic polymorphism parameters and pairwise *r*^2^ values observed in females do not directly indicate stronger SNP–trait associations. Differences in genotype frequencies between sexes may also have affected the statistical power of the association analysis. Therefore, the present results only indicate that significant associations were detected in females within this full-sib family and should not be considered evidence of a female-specific genetic effect. Noncoding variants located in introns or other regulatory regions can potentially affect gene expression or pre-mRNA splicing [35,36]. However, whether such variants within *Mn-PKA-R1* contribute to growth variation in male *M. nipponense* remains unknown and requires further investigation.

Pairwise *r*^2^ values among the seven *Mn-PKA-R1* SNP loci ranged from 0.001 to 0.693 in females and from 0.000 to 0.351 in males. The highest *r*^2^ value in both sexes was observed between C+82748G and A+82750G, reaching 0.693 in females and 0.351 in males.

Within the investigated full-sib family, females exhibited higher pairwise *r*^2^ values and a greater number of relatively correlated SNP pairs than males. No SNP pair exhibited a very high level of LD (*r*^2^ ≥ 0.80) [37]. Because all analyzed individuals were derived from a single full-sib family, the estimated LD is expected to reflect the segregation of a limited set of parental haplotypes within this family and may also be influenced by allele frequencies and finite-sample variation, rather than representing population-wide LD patterns [38,39,40,41]. SNPs associated with economically important traits may have potential value as candidate markers for marker-assisted selection [42]. However, LD patterns and SNP–trait associations estimated from a single full-sib family may not be representative of unrelated breeding populations. Therefore, the four growth-associated SNPs identified in this study should be further validated in independent families, unrelated populations, and different culture environments, together with functional analyses, before their application in breeding programs.

## 4. Materials and Methods

### 4.1. Tissue Collection

All *M. nipponense* specimens used in the present study were obtained from a single culture pond at the Dapu Breeding Base, Wuxi, China (120°13′44″ E, 31°28′22″ N). The individuals used in this study were derived from a single full-sib family. The female and male parents had body weights of 5.32 and 6.59 g, respectively. The parental individuals were not genotyped for the SNP loci identified in the present study. The offspring were hatched in June 2025 and cultured for approximately 5 months. Prior to experimentation, all prawns were acclimatized under controlled laboratory conditions for 3 days, during which water temperature was maintained at 26.0 ± 1.2 °C and dissolved oxygen was kept above 6.0 mg/L. A total of 120 females and 120 males were collected, and body weight, body length, full length, and abdominal width were recorded for each individual. In females, the mean body weight, body length, full length, and abdominal width were 0.935 ± 0.240 g, 29.815 ± 3.093 mm, 43.339 ± 3.526 mm, and 5.437 ± 0.915 mm, respectively. In males, the corresponding values were 2.599 ± 0.930 g, 38.809 ± 4.829 mm, 58.313 ± 7.228 mm, and 7.700 ± 1.001 mm, respectively. Immediately following phenotypic measurement, muscle tissue was dissected from each specimen, snap-frozen in liquid nitrogen to prevent DNA degradation, and stored at −80 °C until DNA extraction.

### 4.2. Genomic DNA Extraction, PCR Amplification, and Sequencing of Mn-PKA-R1

Genomic DNA was extracted from muscle tissue using a Takara DNA extraction kit (Takara Bio Inc., Otsu, Shiga, Japan), according to the manufacturer’s protocol. DNA quality was assessed by 1.2% agarose gel electrophoresis, and DNA concentration was quantified using a UV spectrophotometer (Eppendorf SE, Hamburg, Germany). All DNA samples were stored at −20 °C until further analysis for *Mn-PKA-R1* SNP identification. The *Mn-PKA-R1* genomic sequence of *M. nipponense* deposited in the *M. nipponense* genome database (accession number: GCA_015104395.2) was used for primer design. Primer design was performed using Primer 5.0 (PREMIER Biosoft International, Palo Alto, CA, USA) software (Table 7) [43]. Target DNA fragments were amplified by PCR using specific primers, and the amplification products were examined by 1.2% agarose gel electrophoresis. Purified PCR products were sequenced by Shanghai Shenggong Bioengineering Technology Service Co., Ltd. (Shanghai, China) on an ABI 3730 automated DNA sequencer (Invitrogen Biotechnology Co., Ltd., Carlsbad, CA, USA).

### 4.3. SNP Identification and Association Analysis

Sequence alignments were performed using MEGA software (version 11.0) [44] to detect SNP loci in the coding regions of the *Mn-PKA-R1* gene. The identified SNPs were annotated by aligning the genomic and coding sequences, and translating the corresponding codons. Variants were classified as synonymous or nonsynonymous based on whether the encoded amino acid was altered [45,46]. The effective number of alleles (*N_e_*), observed heterozygosity (*H_o_*), expected heterozygosity (*H_e_*), and Nei’s gene diversity (Nei) were calculated using PopGene32 software (University of Alberta, Edmonton, AB, Canada) [47]. Polymorphic information content (PIC) values were further calculated using the PICcalc program (University of Pannonia, Keszthely, Hungary) [48]. SPSS Statistics (version 27.0; IBM Corp., Armonk, NY, USA) was used to assess the normality of the growth-related traits using the Kolmogorov–Smirnov test [49,50], generate the corresponding normal distribution plots, and analyze the associations between *Mn-PKA-R1* SNP loci and growth traits [51]. Given the pronounced sexual dimorphism in growth reported for *M. nipponense* [19,52], all subsequent correlation and SNP–trait association analyses were performed separately by sex. Pearson correlation analyses among growth-related traits were conducted, and the corresponding correlation heatmaps were generated in R (version 4.5.1) using the ggplot2 package [53]. Associations between SNP genotypes and each growth-related trait were assessed separately in females and males using independent-samples *t*-tests [54]. Differences were regarded as statistically significant at *p* < 0.05.

### 4.4. Linkage Disequilibrium Analysis

Pairwise linkage disequilibrium (LD) among the seven SNP loci identified in the *Mn-PKA-R1* gene was analyzed separately in female and male individuals of *M. nipponense*. Genotype data of the seven SNP loci were converted into PED and INFO files using custom scripts and imported into Haploview version 4.2 (Broad Institute, Cambridge, MA, USA) for LD analysis [55]. Pairwise LD was quantified using the squared correlation coefficient (*r*^2^), which ranges from 0 to 1, with higher values indicating stronger linkage disequilibrium between loci. The pairwise *r*^2^ values generated by Haploview were exported and used to construct LD heatmaps in R (version 4.5.1; R Foundation for Statistical Computing, Vienna, Austria) using the ggplot2 package [53]. The heatmaps were generated separately for the female and male individuals, and the color intensity represented the magnitude of pairwise LD, with darker colors indicating higher *r*^2^ values.

## 5. Conclusions

This study systematically screened SNPs within the coding region of *Mn-PKA-R1* and evaluated their associations with growth-related traits in *M. nipponense*. Within the investigated full-sib family, the mean *N_e_*, *H_o_*, *H_e_*, Nei, and PIC values were higher in females than in males. Four SNPs were significantly associated with growth-related traits only in females within this family. Pairwise *r*^2^ values ranged from 0.001 to 0.693 in females, and from 0.000 to 0.351 in males, with generally higher within-family pairwise *r*^2^ values observed in females. These SNPs represent potential candidate markers for marker-assisted selection, but their associations and breeding value require further validation in additional full-sib families derived from different parental pairs and in unrelated breeding populations.

## Figures and Tables

**Table 1:** The positions of exons of *Mn-PKA-R1*.

Exon	Start	Stop	Length
1	128,346,744	128,346,800	57
2	128,346,920	128,347,012	93
3	128,365,314	128,365,391	78
4	128,366,377	128,366,402	26
5	128,372,771	128,372,793	23
6	128,372,956	128,373,066	111
7	128,409,437	128,409,486	50
8	128,418,656	128,418,715	60
9	128,427,014	128,427,169	156
10	128,428,363	128,428,459	97
11	128,428,833	128,428,890	58
12	128,429,366	128,430,296	931
13	128,431,286	128,431,405	120
14	128,431,513	128,431,681	169
15	128,431,763	128,431,867	105
16	128,432,851	128,433,114	264
17	128,433,277	128,433,425	149
18	128,435,449	128,435,649	201
19	128,438,178	128,438,397	220
20	128,438,950	128,439,153	204
21	128,441,023	128,441,110	88
22	128,443,255	128,443,339	85

**Table 2:** Functional annotation of SNP loci identified in the coding region of *Mn-PKA-R1*.

SNP Locus	Genomic Position	CDS Change	Codon Change	Amino Acid Change	Variant Type
C+82748G	128,429,492	c.936C>G	GAC→GAG	p.Asp312Glu	Nonsynonymous, missense
A+82750G	128,429,494	c.938A>G	CAA→CGA	p.Gln313Arg	Nonsynonymous, missense
A+82766G	128,429,510	c.954A>G	CAA→CAG	p.Gln318=	Synonymous
T+82768G	128,429,512	c.956T>G	GTC→GGC	p.Val319Gly	Nonsynonymous, missense
T+82810G	128,429,554	c.998T>G	GTT→GGT	p.Val333Gly	Nonsynonymous, missense
A+83142G	128,429,886	c.1330A>G	AAT→GAT	p.Asn444Asp	Nonsynonymous, missense
A+83324G	128,430,068	c.1512A>G	GCA→GCG	p.Ala504=	Synonymous

**Table 3:** Genetic polymorphism of SNP loci in the combined sample of *M. nipponense*.

Serial Number	SNP Locus	Effective Number of Alleles (*N_e_*)	Observed Heterozygosity (*H_o_*)	Expected Heterozygosity (*H_e_*)	Nei’s Gene Diversity (Nei)	Polymorphic Information Content (PIC)
1	C+82748G	1.8749	0.7417	0.4676	0.4666	0.3578
2	A+82750G	1.7438	0.6167	0.4274	0.4265	0.3356
3	A+82766G	1.0868	0.0833	0.0800	0.0799	0.0767
4	T+82768G	1.0382	0.0375	0.0369	0.0368	0.0361
5	T+82810G	1.2295	0.2083	0.1870	0.1866	0.1692
6	A+83142G	1.2954	0.2625	0.2285	0.2280	0.2020
7	A+83324G	1.0689	0.0667	0.0646	0.0644	0.0624
	Average	1.3339	0.2881	0.2131	0.2127	0.1771

**Table 4:** Genetic polymorphism of SNP loci in female *M. nipponense*.

Serial Number	SNP Locus	Effective Number of Alleles (*N_e_*)	Observed Heterozygosity (*H_o_*)	Expected Heterozygosity (*H_e_*)	Nei’s Gene Diversity (Nei)	Polymorphic Information Content (PIC)
1	C+82748G	1.7909	0.6583	0.4435	0.4416	0.3441
2	A+82750G	1.6838	0.5667	0.4078	0.4061	0.3236
3	A+82766G	1.1327	0.1250	0.1177	0.1172	0.1103
4	T+82768G	1.0689	0.0667	0.0647	0.0644	0.0624
5	T+82810G	1.4274	0.3667	0.3007	0.2994	0.2546
6	A+83142G	1.3109	0.2750	0.2382	0.2372	0.2091
7	A+83324G	1.0778	0.0750	0.0725	0.0722	0.0696
	Average	1.3561	0.3048	0.2350	0.2340	0.1962

**Table 5:** Genetic polymorphism of SNP loci in male *M. nipponense*.

Serial Number	SNP Locus	Effective Number of Alleles (*N_e_*)	Observed Heterozygosity (*H_o_*)	Expected Heterozygosity (*H_e_*)	Nei’s Gene Diversity (Nei)	Polymorphic Information Content (PIC)
1	C+82748G	1.9406	0.8250	0.4867	0.4847	0.3672
2	A+82750G	1.8000	0.6667	0.4463	0.4444	0.3457
3	A+82766G	1.0425	0.0417	0.0410	0.0408	0.0400
4	T+82768G	1.0084	0.0083	0.0083	0.0083	0.0083
5	T+82810G	1.0512	0.0500	0.0490	0.0488	0.0476
6	A+83142G	1.2800	0.2500	0.2197	0.2188	0.1948
7	A+83324G	1.0600	0.0583	0.0569	0.0566	0.0550
	Average	1.3118	0.2714	0.1868	0.1861	0.1512

**Table 6:** Association analysis between *Mn-PKA-R1* SNP genotypes and growth-related traits in female and male *M. nipponense*.

SNP Locus	Gender	Genotype (Number)	Body Weight (g)	Body Length (mm)	Full Length (mm)	Abdominal Width (mm)
C+82748G	Female	GG:0	/	/	/	/
		CG:79	0.898 ± 0.246 ^a^	29.614 ± 3.194 ^a^	42.968 ± 3.569 ^a^	5.253 ± 0.933 ^a^
		CC:41	1.004 ± 0.213 ^b^	30.202 ± 2.886 ^a^	44.051 ± 3.368 ^a^	5.791 ± 0.771 ^b^
	Male	GG:0	/	/	/	/
		CG:99	2.514 ± 0.868 ^a^	38.430 ± 4.659 ^a^	57.810 ± 6.983 ^a^	7.655 ± 0.974 ^a^
		CC:21	2.999 ± 1.115 ^a^	40.596 ± 5.317 ^a^	60.681 ± 8.043 ^a^	7.909 ± 1.115 ^a^
A+82750G	Female	AA:52	0.989 ± 0.230 ^b^	30.133 ± 2.991 ^a^	43.738 ± 3.397 ^a^	5.734 ± 0.841 ^b^
		AG:68	0.892 ± 0.240 ^a^	29.572 ± 3.168 ^a^	43.032 ± 3.615 ^a^	5.209 ± 0.909 ^a^
		GG:0	/	/	/	/
	Male	AA:40	2.723 ± 1.086 ^a^	39.171 ± 5.357 ^a^	59.323 ± 8.307 ^a^	7.799 ± 1.227 ^a^
		AG:80	2.537 ± 0.842 ^a^	38.628 ± 4.566 ^a^	57.807 ± 6.621 ^a^	7.650 ± 0.869 ^a^
		GG:0	/	/	/	/
A+82766G	Female	AA:105	0.936 ± 0.234 ^a^	29.870 ± 2.970 ^a^	43.316 ± 3.438 ^a^	5.488 ± 0.913 ^a^
		AG:15	0.918 ± 0.282 ^a^	29.431 ± 3.949 ^a^	43.495 ± 4.221 ^a^	5.077 ± 0.868 ^a^
		GG:0	/	/	/	/
	Male	AA:115	2.592 ± 0.942 ^a^	38.769 ± 4.905 ^a^	58.236 ± 7.342 ^a^	7.696 ± 1.018 ^a^
		AG:5	2.766 ± 0.606 ^a^	39.728 ± 2.605 ^a^	60.070 ± 3.664 ^a^	7.778 ± 0.458 ^a^
		GG:0	/	/	/	/
T+82768G	Female	TT:112	0.929 ± 0.241 ^a^	29.786 ± 3.059 ^a^	43.199 ± 3.531 ^a^	5.463 ± 0.917 ^a^
		TG:8	1.000 ± 0.214 ^a^	30.221 ± 3.746 ^a^	45.285 ± 2.984 ^a^	5.063 ± 0.836 ^a^
		GG:0	/	/	/	/
	Male	TT:119	/	/	/	/
		TG:1	/	/	/	/
		GG:0	/	/	/	/
T+82810G	Female	TT:76	0.959 ± 0.220 ^a^	30.045 ± 2.712 ^a^	43.310 ± 3.317 ^a^	5.682 ± 0.819 ^b^
		TG:44	0.892 ± 0.267 ^a^	29.417 ± 3.659 ^a^	43.387 ± 3.899 ^a^	5.013 ± 0.925 ^a^
		GG:0	/	/	/	/
	Male	TT:114	2.608 ± 0.922 ^a^	38.878 ± 4.808 ^a^	58.411 ± 7.201 ^a^	7.720 ± 0.993 ^a^
		TG:6	2.436 ± 1.143 ^a^	37.488 ± 5.498 ^a^	56.443 ± 8.174 ^a^	7.321 ± 1.151 ^a^
		GG:0	/	/	/	/
A+83142G	Female	AA:87	0.949 ± 0.212 ^a^	30.039 ± 2.813 ^a^	43.473 ± 3.244 ^a^	5.577 ± 0.871 ^a^
		AG:33	0.895 ± 0.300 ^a^	29.224 ± 3.714 ^a^	42.981 ± 4.214 ^a^	5.067 ± 0.936 ^a^
		GG:0	/	/	/	/
	Male	AA:90	2.611 ± 0.988 ^a^	38.832 ± 4.991 ^a^	58.252 ± 7.793 ^a^	7.719 ± 1.106 ^a^
		AG:30	2.562 ± 0.742 ^a^	38.740 ± 4.384 ^a^	58.493 ± 5.283 ^a^	7.641 ± 0.587 ^a^
		GG:0	/	/	/	/
A+83324G	Female	AA:111	0.954 ± 0.235 ^b^	29.943 ± 3.024 ^a^	43.647 ± 3.436 ^b^	5.487 ± 0.913 ^b^
		AG:9	0.690 ± 0.148 ^a^	28.238 ± 3.674 ^a^	39.523 ± 2.197 ^a^	4.808 ± 0.710 ^a^
		GG:0	/	/	/	/
	Male	AA:113	2.617 ± 0.929 ^a^	38.915 ± 4.797 ^a^	58.474 ± 7.174 ^a^	7.726 ± 0.997 ^a^
		AG:7	2.314 ± 0.963 ^a^	37.100 ± 5.408 ^a^	55.694 ± 8.165 ^a^	7.272 ± 1.023 ^a^
		GG:0	/	/	/	/

Values are presented as mean ± SD. Within the same sex and trait, values sharing the same lowercase letter are not significantly different, whereas values with different lowercase letters indicate significant differences (*p* < 0.05). Association testing was not performed for T+82768G in males because only one individual carried the TG genotype.

**Table 7:** Primers used in the present study.

Primer	Sequence	Purpose
PKA-SNP-F1	GTCAAGCGTTGGAGGAGGATT	Primers for SNP identification
PKA-SNP-R1	CCAAACGCCATGTCAATTCCA	
PKA-SNP-F2	CACGTCACTTATGCCCACAAA	
PKA-SNP-R2	CTCCCTGACTTTGCTATCACCA	
PKA-SNP-F3	TGAACAAGCGAGAGAGGTGAC	
PKA-SNP-R3	CGGTGTTTCTGTCTTCTCCGA	
PKA-SNP-F4	TCGGAGAAGACAGAAACACCG	
PKA-SNP-R4	CCCTTCACTGCCATCTGAGTT	
PKA-SNP-F5	TCCTATCAACACCGCAGTGAC	
PKA-SNP-R5	CCAACCTTGTGCTTAACAGCC	
PKA-SNP-F6	TTTCGCAGAAAGCAGCAACTC	
PKA-SNP-R6	GTGCGAGCTTGCATAACAACA	

## Data Availability

The original contributions presented in this study are included in the article. Further inquiries can be directed to the corresponding authors.

## References

[1] Fu H., Jiang S., Xiong Y. (2012). Current status and prospects of farming the giant river prawn (*Macrobrachium rosenbergii*) and the oriental river prawn (*Macrobrachium nipponense*) in China. Aquac. Res..

[2] Bureau of Fisheries, Ministry of Agriculture and Rural Affairs, National Fisheries Technology Extension Center, China Society of Fisheries (2026). China Fishery Statistical Yearbook 2026.

[3] Eddine K.D., Gao Z., Daka P., Zhang W., Jiang S., Xiong Y., Qiao H., Fu H. (2025). Evaluation and comparison germplasm resource of two species of Macrobrachium in China (*M. hainanense* and *M. nipponense*). Aquac. Res..

[4] Xue C., Xu K., Jin Y., Bian C., Sun S. (2022). Transcriptome analysis to study the molecular response in the gill and hepatopancreas tissues of *Macrobrachium nipponense* to salinity acclimation. Front. Physiol..

[5] Wu X., Fan Y., Feng J., Ma K., Li J. (2025). Transcriptomic, histological and biochemical analyses of *Macrobrachium nipponense* response to acute heat stress. Aquac. Fish..

[6] Jin S., Bian C., Jiang S., Han K., Xiong Y., Zhang W., Shi C., Qiao H., Gao Z., Li R. (2021). A chromosome-level genome assembly of the oriental river prawn, *Macrobrachium nipponense*. GigaScience.

[7] Gjedrem T. (1983). Genetic variation in quantitative traits and selective breeding in fish and shellfish. Aquaculture.

[8] Olesen I., Gjedrem T., Bentsen H.B., Gjerde B., Rye M. (2003). Breeding programs for sustainable aquaculture. J. Appl. Aquac..

[9] De Verdal H., Komen H., Quillet E., Chatain B., Allal F., Benzie J.A., Vandeputte M. (2018). Improving feed efficiency in fish using selective breeding: A review. Rev. Aquac..

[10] Feng H., Guangwei S., Bo S., Ming Z., Ming L., Yuanyuan Z., Ruibing P. (2022). Current situation and development suggestions of aquaculture commercial breeding in China. J. Fish. Res..

[11] Dunham R. (2023). Aquaculture and Fisheries Biotechnology: Genetic Approaches.

[12] Chavanne H., Janssen K., Hofherr J., Contini F., Haffray P., Komen H., Nielsen E.E., Bargelloni L. (2016). A comprehensive survey on selective breeding programs and seed market in the European aquaculture fish industry. Aquac. Int..

[13] Maqsood H.M., Ahmad S.M. (2017). Advances in molecular markers and their applications in aquaculture and fisheries. Genet. Aquat. Org..

[14] Gopikrishna G., Lakra W.S., Goswami M., Trudeau V.L. (2023). Chapter 4—Application of molecular markers in aquaculture. Frontiers in Aquaculture Biotechnology.

[15] Wang J., Yu X., Chen G., Zhang Y., Tong J. (2022). Molecular characterization of slc5a6a and its association with growth and body conformation in bighead carp (*Hypophthalmichthys nobilis*). Aquac. Rep..

[16] Vignal A., Milan D., SanCristobal M., Eggen A. (2002). A review on SNP and other types of molecular markers and their use in animal genetics. Genet. Sel. Evol..

[17] Yuan J., Yu Y., Zhang X., Li S., Xiang J., Li F. (2023). Recent advances in crustacean genomics and their potential application in aquaculture. Rev. Aquac..

[18] Gao X., Gao Z., Zhang M., Qiao H., Jiang S., Zhang W., Xiong Y., Jin S., Fu H. (2024). Identifying Relationships between Glutathione S-Transferase-2 Single Nucleotide Polymorphisms and Hypoxia Tolerance and Growth Traits in *Macrobrachium nipponense*. Animals.

[19] Jiang S., Xie Y., Gao Z., Niu Y., Ma C., Zhang W., Xiong Y., Qiao H., Fu H. (2024). Studies on the Relationships between Growth and Gonad Development during First Sexual Maturation of *Macrobrachium nipponense* and Associated SNPs Screening. Int. J. Mol. Sci..

[20] Jin S., Lin J., Fu H., Xiong Y., Qiao H., Zhang W., Jiang S. (2026). RNAi Identified the Potential Functions of Actin-like Protein in the Growth Performance of *Macrobrachium nipponense*. Int. J. Mol. Sci..

[21] Zhang Y., Zhang W., Xiong Y., Qiao H., Fu H., Jiang S., Jin S. (2026). Identification of the Potential Functions of the *PKA-R1* Gene in the Regulation of Growth Performance and Molting of *Macrobrachium nipponense* by RNAi. Int. J. Mol. Sci..

[22] Søberg K., Jahnsen T., Rognes T., Skålhegg B.S., Laerdahl J.K. (2013). Evolutionary paths of the cAMP-dependent protein kinase (PKA) catalytic subunits. PLoS ONE.

[23] Knape M.J., Wallbott M., Burghardt N.C.G., Bertinetti D., Hornung J., Schmidt S.H., Lorenz R., Herberg F.W. (2020). Molecular Basis for Ser/Thr Specificity in PKA Signaling. Cells.

[24] Ng P.C., Henikoff S. (2006). Predicting the effects of amino acid substitutions on protein function. Annu. Rev. Genom. Hum. Genet..

[25] Vihinen M. (2015). Types and effects of protein variations. Hum. Genet..

[26] Pudovkin A., Zaykin D., Hedgecock D. (1996). On the potential for estimating the effective number of breeders from heterozygote-excess in progeny. Genetics.

[27] Luikart G., Cornuet J.-M. (1999). Estimating the effective number of breeders from heterozygote excess in progeny. Genetics.

[28] Botstein D., White R.L., Skolnick M., Davis R.W. (1980). Construction of a genetic linkage map in man using restriction fragment length polymorphisms. Am. J. Hum. Genet..

[29] Li M., Liu M., Liu D., Lan X., Lei C., Chen H. (2014). The novel coding region SNPs of PPARGC1A gene and their associations with growth traits in Chinese native cattle. Mol. Biol. Rep..

[30] Xu H., Gui L., Song N., Zhang Y., Wang H., Zan L. (2015). Association of CRTC2 gene polymorphisms with growth and meat quality traits of Qinchuan cattle. Genet. Mol. Res. GMR.

[31] Zhang W., Luo J., Shih Y., Zhang Y., Zhou S., Qin Q., Huang K., Wang Q., Wang X., Ye H. (2025). Incorporating GWAS-derived prior information enhances genomic prediction for body size traits in the mud crab (*Scylla paramamosain*). Comp. Biochem. Physiol. Part D Genom. Proteom..

[32] Duan B., Zhang J., Kang T., Zhang C., Mu S., Guan Y., Ren Y., Li Z., Kang X. (2025). Association analysis reveals SNP markers associated with growth traits in swimming crabs (*Portunus trituberculatus*). Comp. Biochem. Physiol. Part D Genom. Proteom..

[33] Schaid D.J., Chen W., Larson N.B. (2018). From genome-wide associations to candidate causal variants by statistical fine-mapping. Nat. Rev. Genet..

[34] Sauna Z.E., Kimchi-Sarfaty C. (2011). Understanding the contribution of synonymous mutations to human disease. Nat. Rev. Genet..

[35] Cheng F., Zheng W., Liu C., Barbuti P.A., Yu-Taeger L., Casadei N., Huebener-Schmid J., Admard J., Boldt K., Junger K. (2022). Intronic enhancers of the human SNCA gene predominantly regulate its expression in brain in vivo. Sci. Adv..

[36] Jung H., Lee K.S., Choi J.K. (2021). Comprehensive characterisation of intronic mis-splicing mutations in human cancers. Oncogene.

[37] Carlson C.S., Eberle M.A., Rieder M.J., Yi Q., Kruglyak L., Nickerson D.A. (2004). Selecting a Maximally Informative Set of Single-Nucleotide Polymorphisms for Association Analyses Using Linkage Disequilibrium. Am. J. Hum. Genet..

[38] Ding X.D., Simianer H., Zhang Q. (2007). A new method for haplotype inference including full-sib information. Genetics.

[39] Jonker M., van der Vaart A., Bochdanovits Z., Boomsma D. (2011). Estimating and testing linkage disequilibrium from sib data. Stat. Neerl..

[40] VanLiere J.M., Rosenberg N.A. (2008). Mathematical properties of the r2 measure of linkage disequilibrium. Theor. Popul. Biol..

[41] Pe’er I., Chretien Y.R., de Bakker P.I., Barrett J.C., Daly M.J., Altshuler D.M. (2006). Biases and reconciliation in estimates of linkage disequilibrium in the human genome. Am. J. Hum. Genet..

[42] Yue G.H. (2014). Recent advances of genome mapping and marker-assisted selection in aquaculture. Fish Fish..

[43] Lalitha S. (2000). Primer premier 5. Biotech. Softw. Internet Rep..

[44] Tamura K., Stecher G., Kumar S. (2021). MEGA11: Molecular evolutionary genetics analysis version 11. Mol. Biol. Evol..

[45] Den Dunnen J.T., Dalgleish R., Maglott D.R., Hart R.K., Greenblatt M.S., McGowan-Jordan J., Roux A.F., Smith T., Antonarakis S.E., Taschner P.E. (2016). HGVS recommendations for the description of sequence variants: 2016 update. Hum. Mutat..

[46] Plotkin J.B., Kudla G. (2011). Synonymous but not the same: The causes and consequences of codon bias. Nat. Rev. Genet..

[47] Yeh F., Yang R.-C., Boyle T., Ye Z., Xiyan J., Yang R. (2000). POPGENE 32, Microsoft Windows-Based Freeware for Population Genetic Analysis.

[48] Nagy S., Poczai P., Cernák I., Gorji A.M., Hegedűs G., Taller J. (2012). PICcalc: An online program to calculate polymorphic information content for molecular genetic studies. Biochem. Genet..

[49] Berger V.W., Zhou Y. (2014). Kolmogorov–smirnov test: Overview. Wiley Statsref: Statistics Reference Online.

[50] Darling D.A. (1957). The kolmogorov-smirnov, cramer-von mises tests. Ann. Math. Stat..

[51] Mertler C.A., Vannatta R.A., LaVenia K.N. (2021). Advanced and Multivariate Statistical Methods: Practical Application and Interpretation.

[52] Wang M., Jin S., Liu S., Fu H., Zhao Y., Jiang L. (2023). Genome-wide association study of growth and sex traits provides insight into heritable mechanisms underlying growth development of *Macrobrachium nipponense* (Oriental River prawn). Biology.

[53] Wickham H. (2011). ggplot2. Wiley Interdiscip. Rev. Comput. Stat..

[54] Landau S., Everitt B.S. (2003). A Handbook of Statistical Analyses Using SPSS.

[55] Barrett J.C., Fry B., Maller J., Daly M.J. (2005). Haploview: Analysis and visualization of LD and haplotype maps. Bioinformatics.

